# Effects of Common Cooking Methods on Total Mercury and Methylmercury in Sardines and Sprats

**DOI:** 10.3390/foods15162802

**Published:** 2026-08-11

**Authors:** Petr Smid, Lukas Praus, Barbora Lampova, Matyas Orsak, Aneta Kopec, Ivo Doskocil

**Affiliations:** 1 Department of Chemistry, Faculty of Agrobiology, Food and Natural Resources, Czech University of Life Sciences Prague, Kamycka 129, 165 00 Prague, Czech Republic; orsak@af.czu.cz; 2 Department of Agroenvironmental Chemistry and Plant Nutrition, Faculty of Agrobiology, Food and Natural Resources, Czech University of Life Sciences Prague, Kamycka 129, 165 00 Prague, Czech Republic; prausl@af.czu.cz; 3 Department of Microbiology, Nutrition and Dietetics, Faculty of Agrobiology, Food and Natural Resources, Czech University of Life Sciences Prague, Kamycka 129, 165 00 Prague, Czech Republic; lampova@af.czu.cz (B.L.); doskocil@af.czu.cz (I.D.); 4 Department of Human Nutrition and Dietetics, Faculty of Food Technology, University of Agriculture in Kraków, Balicka 122, 30-149 Kraków, Poland; aneta.kopec@urk.edu.pl

**Keywords:** small pelagic fish, sardines, sprats, total mercury, methylmercury, mercury speciation, culinary processing, dietary exposure

## Abstract

The effects of household cooking on mercury speciation in whole small pelagic fish remain insufficiently understood. To address this gap, total mercury (T-Hg), methylmercury (MeHg), and Hg(II) were determined in wet whole-fish homogenates, including viscera, of sardines and sprats before and after boiling, steaming, baking, and frying. T-Hg was determined by AMA-254 and mercury species by HPLC-ICP-MS; results were expressed on wet-weight (ww), dry-weight (dw), and 100 g reference-portion bases. Frying yielded the highest dry matter, underscoring moisture-related differences among reporting bases. T-Hg concentrations ranged from 0.0518 to 0.1322 µg/g dw and MeHg from 0.0273 to 0.0624 µg/g dw. MeHg was detected in all samples, whereas Hg(II) exceeded the method detection limit only in baked and steamed sardines. MeHg amounts per 100 g ranged from 1.38 to 2.35 µg in sardines and from 0.95 to 1.79 µg in sprats. Within the tested batch, no treatment consistently reduced T-Hg or MeHg across both species. By combining analysis of the complete fish matrix with direct mercury speciation and multiple reporting bases, this study provides a food-relevant framework for interpreting mercury in processed small pelagic fish.

## 1. Introduction

Fish and seafood are valued for their nutritional quality and represent important dietary sources of protein, essential trace elements, vitamins, and omega-3 fatty acids [1]. Small pelagic fish such as sardines (*Sardina pilchardus*) and sprats (*Sprattus sprattus*) are important commercial species in Europe and are supplied for human consumption in several product forms. Sardines are widely available as preserved products, whereas sprats are marketed mainly in canned or smoked forms and, to a lesser extent, fresh and whole [2,3,4]. Beyond Europe, the consumption of small fish with bones and organs is also documented in Japan and other regions [5]. Although processing practices vary and not all commercial products retain the viscera, whole or minimally processed small-fish products may retain tissues beyond muscle and therefore represent a distinct matrix for food-safety monitoring and dietary-exposure assessment [6].

Among metallic contaminants, mercury remains a priority because it persists in aquatic environments and is efficiently converted to methylmercury (MeHg), which biomagnifies along aquatic food webs [1]. MeHg is highly bioavailable and toxic, and fish consumption is the dominant pathway of human exposure [7]. At the broader food-system level, international marine-fish trade may redistribute both nutrients and contaminants, including mercury, among consumer populations [8]. In Europe, exposure appraisal and risk characterisation are commonly linked to EFSA’s tolerable weekly intake (TWI) for MeHg (1.3 µg/kg body weight/week, expressed as Hg), highlighting the need for accurate, exposure-relevant characterisation of mercury in commonly consumed fish products [9]. In parallel, Commission Regulation (EU) 2023/915 sets a maximum mercury level of 0.30 mg/kg wet weight for European sprat and sardine or pilchard, with the limit applying to the whole fish when it is intended to be eaten whole [10]. Advances in portable analytical procedures may also support on-site determination and speciation of inorganic Hg and MeHg in fish samples [11].

Published studies evaluating mercury in fish frequently focus on muscle or edible fillet tissue, whereas information on whole-fish matrices and internal organs remains comparatively limited [6,12,13]. This distinction is important because mercury species are not distributed uniformly among fish tissues. MeHg generally represents a higher proportion of T-Hg in muscle, whereas inorganic mercury may contribute relatively more in internal organs. In Baltic sprat, the relative contribution of MeHg was higher in muscle than in whole specimens or the digestive tract, indicating that muscle-only analyses may not adequately represent mercury speciation in fish consumed or processed whole [14]. Beyond tissue type, biological characteristics such as age and body length may also influence muscle Hg concentrations and further complicate comparisons among fish-monitoring studies [15].

Because cooking can markedly modify fish composition, it can also influence how contaminant concentrations are expressed and compared across treatments [6]. Household cooking and other processing steps can influence measured contaminant concentrations by altering moisture and lipid content, even when the contaminant’s absolute mass remains unchanged [16,17]. For mercury in fish, reported post-cooking changes vary across studies and are often difficult to interpret [17]. Dehydration can produce apparent increases in concentration, and results may also be reported on different analytical bases (wet weight versus dry weight) [16,18]. Moreover, many studies report only total mercury, whereas MeHg is toxicologically the most relevant species in fish, and its proportion relative to total mercury strengthens exposure-oriented interpretation [18].

Although cooking-related changes in T-Hg and MeHg concentrations or mercury bioaccessibility have been investigated in fish and seafood [19], evidence specifically addressing mercury speciation after cooking in whole small pelagic fish, rather than in muscle or edible tissue alone, remains limited. To our knowledge, no previous study has combined the direct determination of T-Hg, MeHg, and Hg(II) in wet whole-fish homogenates of sardines and sprats, including the viscera, following boiling, steaming, baking, and frying with parallel interpretation on wet-weight, dry-weight, and 100 g reference-portion bases. This combined approach shows how culinary treatment, whole-fish matrix composition, and reporting basis may jointly influence measured mercury concentrations, apparent speciation profiles, and their exposure-related interpretation, without implying that cooking destroys or chemically transforms mercury. Based on the expected effects of cooking on moisture and matrix composition, we hypothesised that boiling, steaming, baking, and frying would produce species- and treatment-dependent differences in the measured concentrations and apparent speciation profiles of mercury, and that their interpretation would vary according to the reporting basis. The present study, therefore, assessed T-Hg, MeHg, and Hg(II) in raw and cooked whole-fish homogenates of sardines and sprats, with the aim of providing exposure-relevant evidence on how culinary treatment, fish species, and reporting basis influence the interpretation of mercury concentrations and speciation in small pelagic fish products.

## 2. Materials and Methods

### 2.1. Sample Preparation

Whole sardines (*Sardina pilchardus*; country of origin: Morocco, Mediterranean Sea) and sprats (*Sprattus sprattus*; country of origin: Poland, Baltic Sea) were purchased as vacuum-packed products and transported on ice. Fish were processed as whole individuals, including viscera, to reflect products retaining tissues beyond muscle.

For each species, approximately 1 kg of fish from one commercial batch was used to prepare five sample groups: raw control and four common household culinary treatments, namely boiling, steaming, baking, and frying [14], with approximately 200 g of fish assigned to each group. The treatment conditions were as follows: boiling in unsalted water at 100 °C for 10 min; steaming with steam at 100 °C for 20 min; baking in an oven at 180 °C for 20 min; and frying in rapeseed oil at 180 °C for 5 min. A detailed overview of sample preparation and culinary-treatment conditions is provided in Table S1.

After cooking, samples were cooled to room temperature and homogenised. Raw fish samples were homogenised in parallel without thermal treatment. The resulting wet homogenates were used directly for mercury analysis without freeze-drying.

Dry matter content was determined gravimetrically from separate aliquots of the homogenised wet samples associated with the AMA-254 and HPLC-ICP-MS workflows. For each species × culinary treatment × analytical workflow combination, one aliquot was dried at 105 °C in a drying oven (Venticel 111, BMT, Brno, Czech Republic) to constant weight. Dry matter was expressed as a percentage of the original wet sample mass. Mercury concentrations were initially obtained on a wet-weight basis and subsequently normalised to dry weight (µg/g dw) using the corresponding workflow-specific dry matter value. This approach allowed the analysed matrix to reflect the consumed product while enabling comparison among culinary treatments differing in moisture content.

Because one batch per species was used and each treatment was represented by a pooled homogenate, the study should be regarded as a controlled single-batch analytical investigation. Replicate mercury measurements, therefore, represent analytical subsamples rather than biological replicates.

### 2.2. Determination of Total Mercury (T-Hg)

Total mercury (T-Hg) was determined directly in wet whole-fish homogenates using an AMA-254 advanced mercury analyser (Altec Ltd., Prague, Czech Republic).

Approximately 0.03–0.04 g of wet homogenised sample was weighed directly into a nickel combustion boat and analysed without prior digestion or lyophilisation. Ten analytical subsamples were collected from different points of each pooled homogenate and analysed independently. The resulting T-Hg concentrations were initially expressed on a wet-weight basis and subsequently normalised to dry weight using the dry matter content determined for the corresponding AMA-254 workflow aliquot.

ERM-BB186 (European Commission, Joint Research Centre, Geel, Belgium) pig-kidney reference material was included as an auxiliary analytical quality-control material. The assigned Hg value of 0.023 ± 0.011 µg/g dry mass is an information value rather than a certified value. The material was used only as an indicative check of analytical performance, not for calculating method recovery. Because the individual measurement results for the reference material were not available for retrospective evaluation, no measured reference material value or recovery is reported.

### 2.3. Mercury Speciation Analysis

#### 2.3.1. Reagents and Standards

All reagents used for mercury extraction and speciation analysis were of analytical grade or higher purity. The extraction solution consisted of 0.15% (*v*/*v*) 2-mercaptoethanol (≥99%, Carl Roth GmbH, Karlsruhe, Germany) in 0.01 mol/L HCl (Analpure^®^, Analytika Ltd., Prague, Czech Republic), prepared as described by Döker and Bosgelmez [20]. L-cysteine (≥98%, Carl Roth GmbH, Karlsruhe, Germany), HNO_3_ (Analpure^®^, Analytika Ltd., Prague, Czech Republic), ammonium acetate (≥97%, Carl Roth GmbH, Karlsruhe, Germany), methanol (ROTISOLV^®^, Carl Roth GmbH, Karlsruhe, Germany), and acetic acid (Analpure^®^, Analytika Ltd., Prague, Czech Republic) were used for extract dilution and chromatographic separation.

Calibration standards for Hg(II) and methylmercury (MeHg) were prepared from HgCl_2_ (≥99.5%, Honeywell-Fluka, Buchs, Switzerland) and CH_3_HgCl (≥98%, Sigma-Aldrich, Taufkirchen, Germany), respectively.

#### 2.3.2. Extraction Procedure and Optimisation

Mercury species were extracted from homogenised wet whole-fish samples. Approximately 0.6 g of sample was weighed to the nearest 0.0001 g into 15 mL polypropylene centrifuge tubes.

Before routine mercury-speciation analysis, the relative performance of microwave-assisted extraction, rotational shaking, and ultrasound-assisted extraction was compared using baked sardines, boiled sprats, and raw sprats. Three separate analytical aliquots were independently extracted for each matrix–procedure combination. Detailed extraction conditions and the corresponding results are provided in Table S4. Total Hg present in the extracts was determined by ICP-MS under the conditions summarised in Table S5. To minimise mercury memory effects, the rinse procedure described by Li et al. [21] was applied. The comparison evaluated relative extraction performance and did not represent an absolute extraction-recovery study.

Ultrasound-assisted extraction was selected for routine mercury-speciation analysis based on its relative extraction performance and practical suitability. Samples were extracted twice with 6 mL of 0.15% (*v*/*v*) 2-mercaptoethanol in 0.01 mol/L HCl. Each extraction cycle consisted of vortexing for 10 s, sonication in a water bath at 40 °C for 15 min, centrifugation at 2700× *g* for 10 min (C-28A, Boeco, Hamburg, Germany), and decantation of the supernatant. The two supernatants were combined and centrifuged again at 2700× *g* for 10 min.

For each pooled species × culinary treatment homogenate, two separate analytical aliquots were independently weighed, extracted, and analysed using the complete HPLC-ICP-MS workflow.

#### 2.3.3. HPLC-ICP-MS Determination of Mercury Species

Extracts obtained using the selected ultrasound-assisted procedure were diluted 1:2 (*v*/*v*) with the HPLC mobile phase, filtered through a 0.22 µm pore-size filter, and analysed using an Agilent 1260 HPLC system coupled to an Agilent 8900 ICP-MS detector (Agilent Technologies Inc., Santa Clara, CA, USA).

Separation was performed on an Eclipse Plus C18 column (100 × 4.6 mm, 3.5 µm; Agilent Technologies Inc., Santa Clara, CA, USA) under isocratic conditions. The mobile phase, adapted from Jagtap et al. [22], consisted of 0.1% (*v*/*v*) 2-mercaptoethanol, 0.04 mol/L ammonium acetate, and 5% (*v*/*v*) methanol, adjusted to pH 5.4 with acetic acid. The flow rate was 1.2 mL/min, the injection volume was 20 µL, and the column temperature was maintained at 30 °C.

External calibration was performed using individual Hg(II) and MeHg standards prepared at 0.05, 0.25, 1.0, 5.0, and 10 µg/L as Hg. Mercury species were identified by comparing sample retention times with those of the corresponding analytical standards and quantified using species-specific calibration curves. Hg(II) was quantified directly as a separate chromatographic peak and was not calculated by difference from T-Hg and MeHg.

Concentrations of MeHg and Hg(II) were obtained on a wet-weight basis and additionally normalised to dry weight using the corresponding workflow-specific dry matter value.

#### 2.3.4. Analytical Performance

Calibration curves for MeHg and Hg(II) were linear over the investigated concentration range, with coefficients of determination (R^2^) ≥ 0.999. Procedural blanks were below the method detection limits, and no blank correction was applied.

The method limits of detection (LODs), expressed on a wet-weight basis, were 0.003 µg/g ww for Hg(II) and 0.005 µg/g ww for MeHg. A separate limit of quantification was not established. Mercury in Water (SRM 1641d, NIST, Gaithersburg, MD, USA) was used to verify instrumental performance for of Hg determination in solution.

### 2.4. Calculation of Methylmercury Amount per 100 g Reference Portion

The amount of MeHg associated with a standardised 100 g reference portion was calculated by multiplying the wet-weight MeHg concentration (µg/g ww) by 100 g. This reference amount was used to facilitate comparisons among culinary treatments and to support consumer-oriented interpretation; it was not intended to represent actual individual consumption. The resulting values represent the analytically determined amount of MeHg in a 100 g portion and should not be interpreted as estimates of absorbed dose or bioavailability.

### 2.5. Calculation of the Apparent MeHg-to-T-Hg Ratio

For each species × culinary treatment homogenate, the apparent MeHg-to-T-Hg ratio was calculated from the mean dry-matter-normalised concentrations according to the following equation:

$$Apparent\;MeHg\text{-}to\text{-}T\text{-}Hg\;ratio\;\left(\%\right)=\frac{mean\;MeHg\;concentration\;\left(\frac{\mu g}{g}\;dw\right)}{mean\;T\text{-}Hg\;concentration\;\left(\frac{\mu g}{g}\;dw\right)}\times 100$$

Mean T-Hg concentrations were based on ten analytical subsamples, whereas mean MeHg concentrations were based on two independently weighed and extracted analytical aliquots. MeHg and T-Hg were determined using separate analytical workflows, non-paired aliquots, and workflow-specific dry matter values. Aliquot-specific MeHg-to-T-Hg ratios could not be calculated without arbitrarily pairing unrelated analytical results. The ratio of the corresponding mean concentrations was therefore used as a descriptive homogenate-level estimate. The values are reported as apparent ratios only and should not be interpreted as complete mercury-speciation recoveries or mass balances.

### 2.6. Statistical Analysis

Data are expressed as mean ± standard deviation (SD) unless stated otherwise. For total mercury determination, ten analytical subsamples were taken from different points of each homogenised pooled sample and analysed independently (*n* = 10 analytical subsamples). For T-Hg, an exploratory analytical-level comparison was performed separately within each species using the ten analytical subsamples measured for each pooled homogenate. Normality of data distribution was assessed using the Shapiro–Wilk test, whereas homogeneity of variances was evaluated using Levene’s test. Because the assumption of homogeneity of variances was not met, differences among culinary-treatment homogenates were assessed using the non-parametric Kruskal–Wallis test followed by Dunn’s post hoc test with Bonferroni correction. Statistical significance was considered at *p* < 0.05. These comparisons were used only to describe differences among the analysed pooled homogenates at the analytical/subsampling level and were not interpreted as independent biological treatment effects.

For mercury speciation, two separate analytical aliquots of each pooled homogenate were independently weighed, extracted, and analysed by HPLC-ICP-MS. MeHg and Hg(II) results are expressed as mean ± SD of two analytical extractions (*n* = 2). For extraction optimisation, three separate analytical aliquots were weighed and extracted for each matrix–procedure combination. Optimisation results are expressed as mean ± SD of three analytical extractions (*n* = 3) and were evaluated descriptively. No inferential statistical testing was performed for mercury speciation or extraction optimization.

Dry matter content was determined once for each workflow-specific aliquot and is therefore reported as a single value without SD.

Because each species × culinary treatment combination was represented by a single pooled homogenate, replicate mercury measurements represent analytical subsampling or extraction replicates rather than independent biological replicates. Therefore, except for the exploratory analytical-level comparison of T-Hg, the results were interpreted descriptively as within-batch analytical comparisons. No statistical testing was used to draw population-level conclusions regarding the effects of species or culinary treatment.

GraphPad Prism 10.6 was used for data processing and calculation of descriptive statistics, and Statistica 14 for exploratory statistical analysis of T-Hg data. Complete individual analytical measurements for T-Hg and mercury speciation are provided in Tables S2 and S3, respectively.

## 3. Results

### 3.1. Dry Matter Content

Dry matter content varied among species, culinary treatments, and analytical workflows (Table 1 and Table 2). In the AMA-254 workflow used for T-Hg determination, dry matter ranged from 29.2% to 66.9%. In the HPLC-ICP-MS workflow used for mercury speciation, dry matter ranged from 26.2% to 65.7%. These workflow-specific dry matter values were used for dry-weight normalisation of the corresponding mercury concentrations.

Across both workflows, the highest dry matter values were observed after frying, particularly in sprats. In contrast, boiled samples showed comparatively low dry matter values, consistent with the water-based nature of this treatment. These results indicate that culinary processing substantially modified the moisture content of the analysed fish matrix. Therefore, mercury concentrations were interpreted after dry-matter normalisation to support comparison among treatments with different water contents.

### 3.2. Total Mercury Concentrations

Total mercury (T-Hg) concentrations are shown in Table 1. When expressed on a wet-weight basis, T-Hg concentrations ranged from 0.0207 to 0.0408 µg/g ww across all species and treatments. All wet-weight T-Hg concentrations were below the maximum level of 0.30 mg/kg established for sardines and European sprats by Commission Regulation (EU) 2023/915 [10]. After dry-matter normalisation, T-Hg concentrations ranged from 0.0518 to 0.1322 µg/g dw. Exploratory analytical-level statistical comparisons were performed separately within each species and concentration basis using the ten analytical subsamples measured for each pooled homogenate. Significant differences among culinary-treatment homogenates at the analytical/subsampling level are indicated by superscript letters in Table 1.

The T-Hg pattern differed between sardines and sprats. In sardines, dry-matter-normalised T-Hg concentrations ranged from 0.0627 to 0.0930 µg/g dw. The raw sardine sample had the lowest value, whereas the boiled sardine sample had the highest value. Baked, steamed, and fried sardines showed intermediate values of 0.0834, 0.0735, and 0.0723 µg/g dw, respectively. At the analytical/subsampling level, these values were statistically distinguishable among several culinary-treatment homogenates, as shown by the superscript groupings in Table 1.

In sprats, dry-matter-normalised T-Hg concentrations ranged from 0.0518 to 0.1322 µg/g dw. In contrast to sardines, the raw sprat sample had the highest T-Hg concentration, whereas fried sprats had the lowest. Baked, boiled, and steamed sprats showed intermediate values of 0.0592, 0.0710, and 0.0621 µg/g dw, respectively. The analytical-level comparison indicated that raw and fried sprat homogenates differed from several processed samples depending on whether concentrations were expressed on a wet-weight or dry-weight basis.

T-Hg concentrations remained low in all analysed samples. However, the observed patterns were species-specific and depended on culinary treatment and reporting basis. Although statistical differences were identified among some culinary-treatment homogenates at the analytical/subsampling level, each species × treatment combination was represented by a single pooled homogenate. These differences should therefore be interpreted as descriptive within-batch analytical patterns rather than population-level effects of species or culinary treatment.

### 3.3. Mercury Speciation: MeHg and Hg(II)

Preliminary extraction optimisation showed that ultrasound-assisted extraction provided mean extracted-Hg concentrations comparable to rotational shaking in baked sardines and boiled sprats, while a higher mean concentration was obtained using ultrasound-assisted extraction in raw sprats. Microwave-assisted extraction produced lower mean extracted-Hg concentrations than ultrasound-assisted extraction in all three tested matrices. Detailed optimisation results are presented in Table S4.

Mercury speciation results obtained by HPLC-ICP-MS are shown in Table 2, and a representative chromatogram of baked sardines, in which both MeHg and Hg(II) were detected, is provided in Figure S1. Methylmercury (MeHg) was detected in all analysed samples. On a wet-weight basis, MeHg concentrations ranged from 0.0095 to 0.0235 µg/g ww. After dry-matter normalisation using workflow-specific dry matter values, MeHg concentrations ranged from 0.0273 to 0.0624 µg/g dw.

In sardines, dry-matter-normalised MeHg concentrations ranged from 0.0381 to 0.0624 µg/g dw. The lowest value was observed in the raw sample, whereas the highest value was observed in boiled sardines. Baked sardines also showed a comparatively high MeHg concentration, while steamed and fried sardines showed intermediate values.

In sprats, dry-matter-normalised MeHg concentrations ranged from 0.0273 to 0.0390 µg/g dw. The highest value was observed in boiled sprats, whereas the lowest value was observed in fried sprats. Raw, baked, and steamed sprats showed values ranging from 0.0322 to 0.0366 µg/g dw.

Sardines generally showed higher MeHg concentrations than sprats across the corresponding culinary treatments. This pattern was interpreted descriptively because the study was based on pooled homogenates and analytical determinations rather than independent biological replicates.

Hg(II) was below the method LOD in most samples. The method LODs, expressed on a wet-weight basis for the analysed homogenates, were 0.003 µg/g ww for Hg(II) and 0.005 µg/g ww for MeHg. Hg(II) was detected above the method LOD only in baked and steamed sardines, for which measured concentrations of 0.004346 and 0.003070 µg/g ww, respectively, were obtained. In all other samples, Hg(II) was reported as <0.003 µg/g ww. Because a separate LOQ was not established and the steamed sardine value was close to the LOD, these low-level Hg(II) results should be interpreted with caution.

### 3.4. Apparent MeHg-to-T-Hg Ratio

The apparent MeHg-to-T-Hg ratio was calculated as the ratio of the corresponding mean dry-matter-normalised MeHg and T-Hg concentrations obtained using the two analytical workflows (Table 3). Across all samples, the apparent MeHg-to-T-Hg ratio ranged from 25.1% to 67.1%.

The lowest ratio was observed in raw sprats, where the apparent MeHg fraction accounted for 25.1% of T-Hg. In all other samples, the apparent MeHg-to-T-Hg ratio ranged from 52.6% to 67.1%. The highest ratio was observed in boiled sardines. These apparent ratios indicate that MeHg represented a substantial fraction of T-Hg in most analysed samples.

Because T-Hg and MeHg were determined using separate analytical workflows, non-paired aliquots, and workflow-specific dry matter values, these ratios should be interpreted as descriptive homogenate-level estimates rather than as complete mercury mass-balance or speciation recoveries.

### 3.5. Methylmercury Amount per 100 g Reference Portion

The MeHg amount associated with a standardised 100 g reference portion was calculated directly from the wet-weight MeHg concentration. The resulting values are presented in Table 3.

In sardines, the MeHg amount per 100 g serving ranged from 1.38 µg in the raw sample to 2.35 µg in the baked sample. Boiled, steamed, and fried sardines contained 2.04, 1.82, and 1.93 µg MeHg per 100 g reference portion, respectively.

In sprats, the MeHg amount per 100 g reference portion ranged from 0.95 µg in the raw sample to 1.79 µg in the fried sample. Baked, boiled, and steamed sprats contained 1.13, 1.02, and 1.16 µg MeHg per 100 g reference portion, respectively.

These values represent the analytically determined amount of MeHg contained in a standardised 100 g reference portion and do not account for gastrointestinal bioaccessibility or systemic bioavailability.

## 4. Discussion

### 4.1. Effects of Culinary Treatment and Reporting Basis

This study compared T-Hg, MeHg, and Hg(II) in wet whole-fish homogenates of sardines and sprats subjected to four common household culinary treatments. The results showed that culinary processing modified dry matter content and that mercury concentration patterns differed between species, treatments, and reporting bases. Exploratory analytical-level statistical comparisons of T-Hg further indicated differences among some culinary-treatment homogenates; however, these comparisons reflected analytical/subsampling-level differences and were not interpreted as independent biological treatment effects. None of the investigated treatments consistently reduced both T-Hg and MeHg in both species.

The highest dry matter values were observed after frying, particularly in sprats, whereas boiled samples retained comparatively high moisture contents. Similar species- and treatment-dependent changes in the proximate composition of sardines and sprats have been reported previously [6]. These compositional changes are important because concentrations expressed on a wet-weight basis may increase when the cooked product loses water or other matrix constituents, even without a corresponding increase in the absolute amount of mercury.

Ribeiro et al. reported species- and treatment-dependent changes in T-Hg concentrations in cooked predatory fish. Although cooked-product concentrations increased in most species–treatment combinations, correction for raw-to-cooked mass changes produced both net losses and net gains of T-Hg, demonstrating that concentration changes and absolute mercury retention should be interpreted separately [23]. Their results also showed substantial intra-species variability, further supporting the need for caution when generalising the effect of a particular cooking method across different fish matrices. Rodrigues et al. also reported cooking-related differences in T-Hg and MeHg concentrations in fishery products, including sardines. Their findings further support the need to evaluate T-Hg and MeHg separately and to interpret post-cooking concentration changes in relation to changes in product composition and mass rather than as direct evidence of an increase or decrease in the absolute amount of mercury [24].

Perelló et al. investigated the effects of frying, grilling, roasting, and boiling on metal concentrations in several foods, including sardines, hake, and tuna. They observed a general tendency for Hg concentrations in fish to increase after cooking and concluded that culinary processing had only limited value as a means of reducing metal concentrations, with the outcome depending on cooking time, temperature, and medium [25]. These findings are consistent with the present observation that cooking should not be assumed to produce a reliable reduction in the measured Hg concentration of fish.

The simultaneous consideration of wet-weight, dry-weight, and 100 g reference portion values therefore provides complementary information. Wet-weight concentrations describe the product as consumed and are directly relevant to regulatory and dietary exposure assessment. Dry-matter normalisation reduces the immediate influence of variable water content and facilitates comparison of mercury concentrations relative to the solid fraction of the samples. However, it does not correct for cooking-related changes in lipids, proteins, soluble components, or total product yield. Consequently, differences remaining after dry-matter normalisation cannot be attributed to water loss alone. The importance of cooking-related mass loss and reporting basis has also been recognised in MeHg exposure modelling [18].

In sardines, all cooked samples showed higher dry-matter-normalised T-Hg and MeHg concentrations than the raw sample. In sprats, the response was less uniform: raw sprats showed the highest T-Hg concentration on a dry-weight basis, whereas fried sprats showed the lowest dry-matter-normalised T-Hg and MeHg concentrations. The exploratory T-Hg comparisons supported that several of these differences among pooled homogenates were distinguishable at the analytical/subsampling level. However, such contrasting patterns should primarily be interpreted as evidence that the apparent effect of cooking depends on both the treatment and the properties of the fish matrix [23].

The contrasting dry-weight T-Hg patterns may reflect differences in matrix composition and tissue distribution rather than a uniform chemical effect of cooking. Dry-weight normalisation corrects for differences in water content but does not account for cooking-induced losses or gains of other dry-matter components, including proteins, lipids, minerals, and soluble compounds. In sardines, preferential retention of Hg-bearing proteinaceous components, together with the loss of dry-matter components containing comparatively little Hg, may have increased T-Hg concentrations per unit dry mass. Amyot et al. reported that Hg and MeHg tended to remain associated with fish tissue during cooking, whereas several more soluble elements were transferred to cooking juices; their mass-balance results also indicated no substantial change in Hg speciation during cooking [16]. Conversely, the lower dry-weight T-Hg concentrations in cooked sprats may have resulted from the loss of Hg-containing exudates or gastrointestinal material, while frying-oil uptake may have further contributed to dilution on a dry-mass basis. Schmidt et al. similarly reported treatment-dependent losses of Hg species, including losses after frying, without significant interconversion between MeHg and Hg(II) [26]. This interpretation may be particularly relevant to whole sprats because mercury is distributed unevenly among tissues, and the digestive tract of Baltic sprat has been reported to contain higher T-Hg concentrations than the whole specimen or muscle [6]. The comparatively stable MeHg concentrations but lower T-Hg concentrations in cooked sprats further suggest that the observed pattern was not driven solely by MeHg.

The results also demonstrate why dry-weight concentrations should not be interpreted in isolation. Fried sprats showed the lowest MeHg concentration on a dry-weight basis but the highest MeHg amount per 100 g reference portion among the sprat treatments. This reflected the high dry matter content of the fried product, meaning that a 100 g reference portion contained more dry material than an equivalent portion of raw or boiled fish. Similarly, boiled sardines showed the highest MeHg concentration on a dry-weight basis, whereas baked sardines produced the highest 100 g reference-portion MeHg estimate. Reporting basis, therefore, materially affected the interpretation of culinary-treatment effects.

Taken together, the observed post-cooking differences are more plausibly explained by concentration and matrix-composition effects than by demonstrated chemical transformation of mercury species. Moisture loss may increase concentrations expressed on a wet-weight basis, whereas lipid loss or uptake and changes in other dry-matter components may also influence dry-weight values. Because cooking yields, cooking water, frying oil, and released exudates were not analysed, the present study cannot distinguish mercury retention, leaching, redistribution, or transfer into cooking media from true interconversion among mercury species. Accordingly, the observed changes should not be interpreted as evidence of methylation, demethylation, oxidation, reduction, or other chemical transformation during cooking. Such conclusions would require a complete mass-balance design including the cooked product, product yield, and all released cooking media [16,23,26].

### 4.2. Mercury Speciation and Relevance of the Whole-Fish Matrix

MeHg was detected in all analysed samples, whereas Hg(II) was below the method LOD in most cases and was detected above the method LOD only in baked and steamed sardines. This finding underscores the merit of determining MeHg directly rather than relying exclusively on T-Hg. However, the absence of Hg(II) above the method LOD should not be interpreted as evidence that all remaining mercury was present as MeHg. Other unquantified Hg species, incomplete extraction, and analytical differences between the T-Hg and speciation workflows may contribute to the discrepancy between MeHg and T-Hg.

Foundational studies provide further context for the interpretation of mercury speciation in fish tissue. Bloom found monomethylmercury to be the predominant measured mercury form in edible fish muscle and also highlighted the influence of analytical variability and sample homogenisation on calculated MeHg-to-T-Hg proportions [27]. Harris et al. subsequently demonstrated that tissue-bound methylmercury in fish is coordinated to a sulfur donor in a thiolate environment resembling cysteine [28]. These findings support both the predominance and strong tissue association of MeHg in fish muscle but do not imply that muscle-based MeHg-to-T-Hg proportions are directly transferable to complete fish homogenates containing the viscera.

The apparent MeHg-to-T-Hg ratio ranged from 25.1% to 67.1%. Except for raw sprats, the apparent ratios ranged from 52.6% to 67.1%, suggesting that MeHg represented a substantial proportion of T-Hg in most analysed homogenates.

The apparent ratios observed in the present study were mostly at or below the lower end of the 66.7–92.9% range reported by Barone et al. for fish muscle [29]. In that study, European pilchard muscle contained approximately 0.03 µg/g ww T-Hg and 0.02 µg/g ww MeHg. These findings support the expectation that MeHg constitutes a major fraction of mercury in muscle tissue but also indicate that muscle-based ratios may not be directly transferable to whole-fish matrices.

Rodrigues et al. (2023) reported a MeHg-to-T-Hg ratio of 0.43 for grilled sardines, which falls within the range observed in the present study and is lower than the muscle-based range reported by Barone et al. This comparison further indicates that MeHg should not automatically be assumed to account for nearly all T-Hg in cooked sardine products, although differences in sample matrix and analytical design limit direct numerical comparison [24].

Not all commercial sardine and sprat products retain the viscera; yet, analysis of the entire fish provides a broader food-safety scenario for products consumed or processed with tissues beyond isolated muscle. Inclusion of viscera may also provide a plausible explanation for the comparatively low apparent MeHg proportions observed in the present study, because mercury species are not distributed uniformly among fish tissues.

In Baltic sprat, Polak-Juszczak reported MeHg proportions of 63.0% in muscle, 44.4% in whole individuals, and 22.7% in the digestive tract [6]. This tissue-dependent gradient supports the interpretation that inclusion of viscera can reduce the MeHg fraction relative to muscle-only samples. Nevertheless, direct numerical comparison remains limited because inorganic mercury was calculated as the difference between T-Hg and MeHg in that study, whereas Hg(II) was chromatographically separated and quantified directly in the present study.

This matrix effect may be relevant to the particularly low apparent MeHg-to-T-Hg ratio calculated for raw sprats. However, the value of 25.1% should not be interpreted as proof that the remaining mercury was present as inorganic Hg, because Hg(II) was below the wet-weight LOD in this sample. In addition, T-Hg and MeHg were determined using separate analytical workflows, non-paired aliquots, different numbers of analytical replicates, and workflow-specific dry matter values. Because the measurements were not paired, aliquot-specific ratios could not be calculated without arbitrarily pairing unrelated analytical results. The ratios of the corresponding mean concentrations should therefore be interpreted as descriptive, apparent homogenate-level estimates rather than as complete speciation recoveries or mercury mass balances.

### 4.3. Comparison with Previously Reported Mercury Concentrations

The raw sardine homogenate contained 0.0228 µg/g ww T-Hg and 0.0138 µg/g ww MeHg. These concentrations were slightly lower than, but of the same order of magnitude as, concentrations previously reported for European pilchard muscle or flesh. Barone et al. reported approximately 0.03 µg/g ww T-Hg and 0.02 µg/g ww MeHg [29], whereas Mehouel et al. reported mean concentrations of approximately 0.04 µg/g ww for both T-Hg and MeHg in sardine flesh collected from the Algerian coast [30].

Although these studies provide useful concentration benchmarks, direct comparison is limited by differences in geographic origin, fish size, season, sampling strategy, analytical procedure, and analysed matrix. Both cited studies primarily analysed muscle or flesh, whereas the present study used homogenates of whole fish, including viscera. Mehouel et al. also evaluated mercury exposure using assumptions specific to adult consumers in Algeria; their risk conclusions should therefore not be transferred directly to the present dataset [30].

Raw sprats contained more T-Hg than raw sardines on both wet- and dry-weight bases but less MeHg. This divergence illustrates why T-Hg may not always serve as a reliable proxy for MeHg in whole-fish matrices. Previous research on Baltic fish has shown that mercury concentrations and speciation vary with trophic position, age, size, feeding ecology, geographic area, and analysed tissue [6].

The sardines and sprats investigated in the present study also originated from different marine regions—the Mediterranean Sea and the Baltic Sea, respectively. At a broader scale, the UNEP Global Mercury Assessment documents worldwide mercury emissions and releases, its transport through atmospheric and aquatic systems, and its occurrence in biota [31]. The observed differences cannot be attributed to species identity alone. They may reflect a combination of species, origin, biological characteristics, environmental exposure, and matrix composition. Within the investigated batches, however, MeHg concentrations and 100 g reference-portion estimates were generally higher in sardines than in sprats, whereas T-Hg patterns were less consistent. These findings should be regarded as batch-specific observations that require confirmation with independently collected samples from multiple origins and seasons.

### 4.4. Regulatory and Dietary Exposure Relevance

Wet-weight T-Hg concentrations ranged from 0.0207 to 0.0408 mg/kg and were therefore below the EU maximum level of 0.30 mg/kg applicable to sardines and European sprats [10]. The measured concentrations ranged from approximately 6.9% to 13.6% of the regulatory maximum, with the highest value observed in raw sprats. Thus, all raw and processed samples analysed in the present study remained well below the applicable maximum level. EU legislation specifies that the maximum level is expressed on a wet-weight basis and that, when fish are intended to be eaten whole, the maximum level applies to the whole fish [10]. Analysis of wet homogenates containing all tissues is therefore directly relevant to the regulatory interpretation of small pelagic products consumed or processed with tissues beyond muscle. Still, this comparison applies only to the investigated batches and should not be interpreted as evidence of general market-wide compliance for these species.

Still, concentrations below the regulatory maximum do not imply the absence of dietary exposure. The MeHg amount in a standardised 100 g reference portion ranged from 1.38 to 2.35 µg for sardines and from 0.95 to 1.79 µg for sprats. The toxicological relevance of these amounts depends on body weight, consumption frequency, exposure from other foods, and consumer susceptibility.

EFSA established a tolerable weekly intake of 1.3 µg MeHg/kg body weight per week, expressed as Hg, with particular concern for neurodevelopmental effects [9]. The recent Joint FAO/WHO Expert Consultation further emphasised that fish-consumption risk–benefit assessment should account for local consumption habits, fish contamination levels, nutrient content, nutritional status, cultural practices, and population characteristics [32]. The 100 g reference-portion values reported here should therefore be interpreted as standardised inputs to dietary exposure assessment rather than as standalone estimates of individual risk or benefit.

Under the tested conditions, none of the culinary treatments consistently reduced MeHg across both species and reporting bases. Culinary method alone should therefore not be considered a dependable exposure-reduction strategy. Species, portion size, consumption frequency, and exposure from other foods remain relevant considerations in dietary exposure assessment.

The present study assessed mercury concentration but not gastrointestinal bioaccessibility or systemic bioavailability. Cooking may modify interactions between mercury species and proteins or other matrix components without producing a proportional change in total concentration.

In vitro studies indicate that cooking may alter mercury bioaccessibility, although the direction and magnitude of the effect depend on the fish matrix, cooking conditions, mercury endpoint, and digestion model. In Pontic shad, Milea et al. (2023) reported lower Hg bioaccessibility after baking and frying than in raw fish [33]. Similarly, Packull-McCormick et al. (2023) found that pan-frying freshwater fish increased measured T-Hg concentrations, likely because of moisture loss, while reducing bioaccessible T-Hg concentrations [34]. Together, these findings demonstrate that cooking-related changes in analytical concentration and gastrointestinal bioaccessibility may proceed in opposite directions. Changes in MeHg bioaccessibility have also been reported in other fish matrices [19]. Because in vitro bioaccessibility does not necessarily predict systemic bioavailability, no bioaccessibility correction was applied to the 100 g reference-portion estimates in the present study.

The reference-portion estimates should therefore be interpreted as concentration-based exposure values. They quantify the amount of analytically determined MeHg present in a standartised 100 g reference portion but do not represent actual individual intake or predict the fraction absorbed by an individual consumer.

### 4.5. Strengths, Limitations, and Future Research

A major strength of this study is the combined determination of T-Hg, MeHg, and directly quantified Hg(II) in wet homogenates prepared from entire sardines and sprats exposed to four common household culinary treatments. Analysis of the wet matrix increased the relevance of the findings to the product as consumed, while workflow-specific dry matter measurements enabled comparison among samples with different moisture contents. The use of 100 g reference-portion MeHg estimates further connected analytical concentrations with consumer-oriented exposure interpretation.

The study nevertheless has important limitations. The study included only two small pelagic fish species and one commercial batch per species. Each species–treatment combination was represented by one pooled homogenate. The ten AMA subsamples and two analytical extractions for mercury speciation therefore characterised analytical and subsampling variability rather than biological variability. Accordingly, the exploratory statistical comparisons performed for T-Hg should be interpreted only at the analytical/subsampling level and cannot be used to infer population-level treatment effects. The observed patterns cannot be extrapolated to broader sardine and sprat populations or generalised to other fish groups. They are also not directly transferable to cephalopods, which represent biologically and compositionally distinct seafood matrices.

Use of the whole-fish matrix increases relevance to products consumed whole but limits direct comparison with studies based on fillets or muscle. Cooking yields were not recorded, and mercury concentrations and speciation were not determined in boiling water, frying oil, or released exudates. It was not possible to establish mercury retention factors, evaluate transfer of individual mercury species into cooking media, or construct complete mass balances.

T-Hg and mercury species were also determined using separate analytical workflows, non-paired aliquots, and different numbers of analytical replicates. The apparent MeHg-to-T-Hg ratios were therefore calculated from the corresponding mean concentrations and should be interpreted only as descriptive homogenate-level estimates, not as validated speciation recoveries or mercury mass balances.

A further limitation is that no matrix-matched certified reference material or species-specific recovery data were available for the complete extraction and speciation workflow. The solution-based reference material was used as an instrumental performance check but did not validate extraction recovery from the fish matrix.

Future studies should include a broader range of fish and selected cephalopod species, multiple independent batches, matched raw and cooked samples, measurement of cooking yield, analysis of cooking media, matrix-matched reference materials or species-specific spike-recovery experiments, and evaluation of MeHg bioaccessibility.

Overall, the observed patterns were specific to the investigated species, batches, and matrices and varied according to whether mercury concentrations were expressed on a wet-weight, dry-weight, or 100 g reference-portion basis. These findings highlight the importance of mercury speciation, characterisation of the complete fish matrix, and consistent reporting when interpreting mercury data for processed small pelagic fish.

## 5. Conclusions

Common household culinary treatments did not consistently reduce T-Hg or MeHg concentrations in whole-fish homogenates of sardines and sprats. The direction and magnitude of the observed differences varied between species and depended on whether the results were expressed on a wet-weight, dry-weight, or 100 g reference-portion basis. Within the investigated batches, sardines showed higher MeHg concentrations and MeHg amounts per 100 g reference portion than sprats across all treatments, whereas T-Hg patterns were less consistent. All wet-weight T-Hg concentrations remained below the EU maximum level of 0.30 mg/kg applicable to these species.

From an exposure-assessment perspective, cooking should therefore not be assumed to reduce mercury intake. Moisture loss and other cooking-related changes in matrix composition may alter measured concentrations, and a lower dry-weight concentration does not necessarily correspond to a lower MeHg amount per 100 g reference portion. Assessment of these cooked fish products should therefore consider product-as-consumed concentrations, actual portion size, fish species, and reporting basis together. Because only one pooled commercial batch per species was examined, these findings should be regarded as batch-specific and confirmed in studies using independent biological batches.

## Figures and Tables

**Table 1:** Total mercury determined by AMA-254.

Species	Treatment	Dry Matter Used for T-Hg Normalisation (%)	T-Hg (µg/g ww ^1^)	T-Hg (µg/g dw ^2^)
Sardines	Raw	36.30	0.0228 ± 0.0011 ^c^	0.0627 ± 0.0031 ^c^
	Baked	44.06	0.0367 ± 0.0014 ^ab^	0.0834 ± 0.0031 ^ab^
	Boiled	33.49	0.0311 ± 0.0017 ^c^	0.0930 ± 0.0050 ^a^
	Steamed	43.41	0.0319 ± 0.0008 ^bc^	0.0735 ± 0.0019 ^bc^
	Fried	51.74	0.0374 ± 0.0009 ^a^	0.0723 ± 0.0017 ^bc^
Sprats	Raw	30.85	0.0408 ± 0.0072 ^a^	0.1322 ± 0.0233 ^a^
	Baked	37.60	0.0223 ± 0.0024 ^b^	0.0592 ± 0.0063 ^ab^
	Boiled	29.22	0.0207 ± 0.0019 ^b^	0.0710 ± 0.0066 ^bc^
	Steamed	34.29	0.0213 ± 0.0009 ^b^	0.0621 ± 0.0026 ^bc^
	Fried	66.93	0.0347 ± 0.0011 ^a^	0.0518 ± 0.0016 ^c^

Dry matter values correspond to aliquots used in the AMA-254 workflow. Values are expressed as mean ± SD of ten analytical subsamples. Different superscript letters within the same species and concentration basis indicate statistically significant differences among culinary-treatment homogenates at the analytical/subsampling level only (Kruskal–Wallis test followed by Dunn’s post hoc test with Bonferroni correction, *p* < 0.05). Because each species × culinary treatment combination was represented by a single pooled homogenate, these comparisons do not represent independent biological treatment effects and should be interpreted as descriptive within-batch analytical comparisons.; ^1^ ww—wet weight, ^2^ dw—dry weight.

**Table 2:** Mercury speciation determined by HPLC-ICP-MS.

Species	Treatment	Dry Matter Used for Speciation Normalisation (%)	MeHg (µg/g ww ^1^)	MeHg (µg/g dw ^2^)	Hg(II) (µg/g ww ^1^)	Hg(II) (µg/g dw ^2^)
Sardines	Raw	36.30	0.0138 ± 0.0010	0.0381 ± 0.0027	<0.003	-
	Baked	43.65	0.0235 ± 0.0014	0.0539 ± 0.0031	0.004346 ± 0.000026	0.009956 ± 0.000060
	Boiled	32.70	0.0204 ± 0.0006	0.0624 ± 0.0020	<0.003	-
	Steamed	41.20	0.0182 ± 0.0023	0.0441 ± 0.0055	0.003070 ± 0.000003	0.007450 ± 0.000007
	Fried	48.45	0.0193 ± 0.0009	0.0398 ± 0.0018	<0.003	-
Sprats	Raw	28.61	0.0095 ± 0.0011	0.0331 ± 0.0038	<0.003	-
	Baked	34.99	0.0113 ± 0.0008	0.0322 ± 0.0024	<0.003	-
	Boiled	26.18	0.0102 ± 0.0013	0.0390 ± 0.0048	<0.003	-
	Steamed	31.74	0.0116 ± 0.0004	0.0366 ± 0.0011	<0.003	-
	Fried	65.69	0.0179 ± 0.0019	0.0273 ± 0.0029	<0.003	-

Dry matter values correspond to aliquots used in the HPLC-ICP-MS speciation workflow. MeHg and Hg(II) values are expressed as mean ± SD of two independent analytical extractions from separate aliquots of each pooled whole-fish homogenate (*n* = 2). These replicates do not represent independent biological samples. Method LODs were expressed on a wet-weight basis: 0.003 µg/g ww for Hg(II) and 0.005 µg/g ww for MeHg. Values below the Hg(II) LOD are reported as <0.003 µg/g ww. Dashes indicate that dry-weight-normalised Hg(II) values were not calculated for samples below the wet-weight LOD; ^1^ ww—wet weight, ^2^ dw—dry weight.

**Table 3:** Apparent MeHg-to-T-Hg ratio and MeHg amount per 100 g reference portion.

Species	Treatment	T-Hg (µg/g dw *)	MeHg (µg/g dw *)	Apparent MeHg/T-Hg (%)	MeHg Amount per 100 g Reference Portion (µg)
Sardines	Raw	0.0627	0.0381	60.8	1.38
	Baked	0.0834	0.0539	64.7	2.35
	Boiled	0.0930	0.0624	67.1	2.04
	Steamed	0.0735	0.0441	60.1	1.82
	Fried	0.0723	0.0398	55.1	1.93
Sprats	Raw	0.1322	0.0331	25.1	0.95
	Baked	0.0592	0.0322	54.5	1.13
	Boiled	0.0710	0.0390	55.0	1.02
	Steamed	0.0621	0.0366	58.9	1.16
	Fried	0.0518	0.0273	52.6	1.79

Apparent MeHg-to-T-Hg ratios were calculated as ratios of the corresponding mean dry-matter-normalised concentrations obtained using separate analytical workflows and non-paired aliquots. The values represent descriptive homogenate-level estimates and should not be interpreted as complete mercury mass-balance or speciation recoveries; * dw—dry weight.

## Data Availability

The raw data supporting the conclusions of this article will be made available by the authors on request. In addition, the data presented in this study are openly available in the University of Agriculture in Krakow Research Data Repository at: https://repo.ur.krakow.pl/info/researchdata/URK5407808d5a094203953cf6176358b11b/ (accessed on 3 August 2026).

## Supplementary Materials

The following supporting information can be downloaded at: https://www.mdpi.com/article/10.3390/foods15162802/s1, Table S1: Detailed conditions used for the culinary treatment of sardines and sprats; Table S2: Individual total mercury results obtained by AMA-254 from pooled whole-fish homogenates; Table S3: Individual MeHg and Hg(II) results obtained by HPLC-ICP-MS from pooled whole-fish homogenates; Table S4: Comparison of the relative extraction performance of three procedures in selected fish matrices; Table S5: ICP-MS conditions used to evaluate the relative extraction performance of the tested procedures; Figure S1: Representative HPLC–ICP-MS chromatogram of mercury species in baked sardines.

## Abbreviations

The following abbreviations are used in this manuscript:

TWI	Tolerable weekly intake
LOD	Limit of detection
ww	Wet weight
dw	Dry weight
