# Supplementary material for: Effects of Common Cooking Methods on Total Mercury and Methylmercury in Sardines and Sprats
Source: Foods. 2026 Aug 11;15(16):2802. doi: 10.3390/foods15162802 (PMC13512054; doi:10.3390/foods15162802)
Supplement: Supplementary file 1 [file foods-15-02802-s001.zip › foods-4439680-supplementary.pdf]

Effects of Common Cooking Methods on Total Mercury and Methylmercury in Sardines and Sprats

Smid P [1,*], Praus L [2], Lampova B [3], Orsak M [1], Kopec A [4] and Doskocil I [3]

[1] Department of Chemistry, Faculty of Agrobiology, Food and Natural Resources, Czech University of Life Sciences Prague, Kamycka 129, 165 00 Prague, Czech Republic; email: smidp@af.czu.cz, orsak@af.czu.cz
[2] Department of Agroenvironmental Chemistry and Plant Nutrition, Faculty of Agrobiology, Food and Natural Resources, Czech University of Life Sciences Prague, Kamycka 129, 165 00 Prague, Czech Republic; email: prausl@af.czu.cz
[3] Department of Microbiology, Nutrition and Dietetics, Faculty of Agrobiology, Food and Natural Resources, Czech University of Life Sciences Prague, Kamycka 129, 165 00 Prague, Czech Republic; emails: lampova@af.czu.cz, doskocil@af.czu.cz
[4] Department of Human Nutrition and Dietetics, Faculty of Food Technology, University of Agriculture in Kraków, Balicka 122, 30-149 Kraków, Poland; email: aneta.kopec@urk.edu.pl
* Correspondence: smidp@af.czu.cz

Table S1: Detailed conditions used for the culinary treatment of sardines and sprats

Treatment	Temperature	Duration	Cooking medium	Equipment	Operational details
**Baking**	180 °C	20 min	None	Domestic pre-heated oven	Whole fish placed in a glass baking dish covered with aluminium foil
**Boiling**	100 °C	10 min	Unsalted water	Domestic boiling equipment	Whole fish immersed in boiling water
**Steaming**	100 °C	20 min	Steam	Steamer	Whole fish exposed to steam
**Frying**	180 °C	5 min	Rapeseed oil	Frying pan	Whole fish pan-fried

Note: Approximately 200 g of whole fish, including viscera, was allocated to each treatment group for each species.

Table S2: Individual total mercury results obtained by AMA-254 from pooled whole-fish homogenates

Species	Treatment	Individual T-Hg measurements (µg/g ww[1]; *n* = 10)	Dry matter (%)	T-Hg (µg/g ww[1]), mean ± SD	T-Hg (µg/g dw[2]), mean ± SD
Sardine	Raw	0.023867; 0.021488; 0.023450; 0.023190; 0.022821; 0.023974; 0.021020; 0.021060; 0.023227; 0.023428	36.30	0.0228 ± 0.0011c	0.0627 ± 0.0031c
	Baked	0.035571; 0.035443; 0.035305; 0.035017; 0.037669; 0.037689; 0.036260; 0.037517; 0.038270; 0.038664	44.06	0.0367 ± 0.0014ab	0.0834 ± 0.0031ab
	Boiled	0.029130; 0.030973; 0.033443; 0.033495; 0.031737; 0.030327; 0.029633; 0.032929; 0.030410; 0.029313	33.49	0.0311 ± 0.0017c	0.0930 ± 0.0050a
	Steamed	0.030692; 0.031317; 0.032484; 0.031567; 0.031368; 0.030880; 0.032940; 0.032306; 0.032655; 0.032690	43.41	0.0319 ± 0.0008bc	0.0735 ± 0.0019bc
	Fried	0.037052; 0.037940; 0.036728; 0.038425; 0.037160; 0.038806; 0.037616; 0.037190; 0.035783; 0.037411	51.74	0.0374 ± 0.0009a	0.0723 ± 0.0017bc
Sprat	Raw	0.050782; 0.040802; 0.048559; 0.049360; 0.035500; 0.032070; 0.044480; 0.039340; 0.032180; 0.034753	30.85	0.0408 ± 0.0072a	0.1322 ± 0.0233a
	Baked	0.025120; 0.027000; 0.023220; 0.022225; 0.019173; 0.019643; 0.020898; 0.021420; 0.021620; 0.022204	37.60	0.0223 ± 0.0024b	0.0592 ± 0.0063ab
	Boiled	0.019490; 0.019066; 0.020442; 0.021510; 0.023760; 0.020960; 0.023700; 0.021420; 0.018785; 0.018233	29.22	0.0207 ± 0.0019b	0.0710 ± 0.0066bc
	Steamed	0.022028; 0.020158; 0.021959; 0.020919; 0.022439; 0.021680; 0.022001; 0.021690; 0.020147; 0.020060	34.29	0.0213 ± 0.0009b	0.0621 ± 0.0026bc
	Fried	0.035280; 0.035616; 0.035384; 0.033251; 0.034262; 0.033270; 0.034860; 0.033494; 0.034900; 0.036440	66.93	0.0347 ± 0.0011a	0.0518 ± 0.0016c

Note: Ten analytical subsamples were taken from different locations of each pooled wet whole-fish homogenate and analysed independently by AMA-254. Means and sample standard deviations were calculated using n − 1 in the denominator (STDEV.S). These measurements characterise analytical and within-homogenate subsampling variability and do not represent independent biological replicates. Dry-weight values were calculated using the dry matter content determined for the corresponding AMA-254 workflow aliquot. Superscript letters assigned to mean values correspond to the exploratory analytical-level comparison reported in Table 1. Different letters within the same species and concentration basis indicate statistically significant differences among culinary-treatment homogenates at the analytical/subsampling level only and do not represent independent biological treatment effects.[1]ww – Wet weight, [2]dw – Dry weight.

Table S3: Individual MeHg and Hg(II) results obtained by HPLC-ICP-MS from pooled whole-fish homogenates

Species	Treatment	Dry matter (%)	MeHg A (µg/g ww[1])	MeHg B (µg/g ww[1])	MeHg (µg/g ww[1]), mean ± SD	MeHg (µg/g dw[2]), mean ± SD	Hg(II) A (µg/g ww[1])	Hg(II) B (µg/g ww[1])	Hg(II) (µg/g ww[1]), mean ± SD	Hg(II) (µg/g dw[2]), mean ± SD
**Sardine**	Raw	36.30	0.014542	0.013141	0.0138 ± 0.0010	0.0381 ± 0.0027	<0.003	<0.003	<0.003	—
**Sardine**	Baked	43.65	0.022572	0.024509	0.0235 ± 0.0014	0.0539 ± 0.0031	0.004364	0.004327	0.004346 ± 0.000026	0.009956 ± 0.000060
**Sardine**	Boiled	32.70	0.019938	0.020854	0.0204 ± 0.0006	0.0624 ± 0.0020	<0.003	<0.003	<0.003	—
**Sardine**	Steamed	41.20	0.019801	0.016569	0.0182 ± 0.0023	0.0441 ± 0.0055	0.003067	0.003072	0.003070 ± 0.000003	0.007450 ± 0.000007
**Sardine**	Fried	48.45	0.018669	0.019908	0.0193 ± 0.0009	0.0398 ± 0.0018	<0.003	<0.003	<0.003	—
**Sprat**	Raw	28.61	0.008710	0.010255	0.0095 ± 0.0011	0.0331 ± 0.0038	<0.003	<0.003	<0.003	—
**Sprat**	Baked	34.99	0.011877	0.010676	0.0113 ± 0.0008	0.0322 ± 0.0024	<0.003	<0.003	<0.003	—
**Sprat**	Boiled	26.18	0.011105	0.009314	0.0102 ± 0.0013	0.0390 ± 0.0048	<0.003	<0.003	<0.003	—
**Sprat**	Steamed	31.74	0.011363	0.011859	0.0116 ± 0.0004	0.0366 ± 0.0011	<0.003	<0.003	<0.003	—
**Sprat**	Fried	65.69	0.019284	0.016551	0.0179 ± 0.0019	0.0273 ± 0.0029	<0.003	<0.003	<0.003	—

Note: MeHg and Hg(II) were determined from two separate analytical aliquots of each pooled whole-fish homogenate. Each aliquot was independently weighed, extracted, and analysed. Results are expressed as mean ± sample SD (n = 2 analytical extractions). Means and sample SDs were calculated using n − 1 in the denominator. Method LODs, expressed on a wet-weight basis, were 0.005 µg/g ww for MeHg and 0.003 µg/g ww for Hg(II). A separate LOQ was not established. Results below the Hg(II) LOD were reported as <0.003 µg/g ww and were not converted to dry weight. Dry-weight values were calculated using the dry matter content determined for the corresponding speciation-workflow aliquot. These analytical extractions do not represent independent biological replicates. Hg(II) means and SDs are displayed to six decimal places.[1]ww – Wet weight, [2]dw – Dry weight.

Table S4: Comparison of the relative extraction performance of three procedures in selected fish matrices

Matrix	Extraction procedure	Replicate 1 (µg/g dw[1])	Replicate 2 (µg/g dw[1])	Replicate 3 (µg/g dw[1])	Extracted Hg (µg/g dw[1]), mean ± SD	Extraction conditions
**Baked sardines**	Rotational shaking	0.06197	0.06506	0.06537	0.0641 ± 0.0019	12 mL extractant; vortexing for 10 s; 30 rpm for 12 h at 22 ± 1 °C
**Baked sardines**	Ultrasound-assisted	0.06566	0.06466	0.06326	0.0645 ± 0.0012	2 × 6 mL extractant; vortexing for 10 s; sonication at 40 °C for 15 min per cycle; centrifugation at 2700 × g for 10 min
**Baked sardines**	Microwave-assisted	0.06245	0.05331	0.05548	0.0571 ± 0.0048	12 mL extractant; vortexing for 10 s; 60 °C for 2 min with a 1 min ramp
**Boiled sprats**	Rotational shaking	0.05237	0.05240	0.04897	0.0512 ± 0.0020	12 mL extractant; vortexing for 10 s; 30 rpm for 12 h at 22 ± 1 °C
**Boiled sprats**	Ultrasound-assisted	0.05286	0.04931	0.05238	0.0515 ± 0.0019	2 × 6 mL extractant; vortexing for 10 s; sonication at 40 °C for 15 min per cycle; centrifugation at 2700 × g for 10 min
**Boiled sprats**	Microwave-assisted	0.04338	0.04913	0.04520	0.0459 ± 0.0029	12 mL extractant; vortexing for 10 s; 60 °C for 2 min with a 1 min ramp
**Raw sprats**	Rotational shaking	0.03318	0.03336	0.03175	0.0328 ± 0.0009	12 mL extractant; vortexing for 10 s; 30 rpm for 12 h at 22 ± 1 °C
**Raw sprats**	Ultrasound-assisted	0.04170	0.04444	0.03932	0.0418 ± 0.0026	2 × 6 mL extractant; vortexing for 10 s; sonication at 40 °C for 15 min per cycle; centrifugation at 2700 × g for 10 min
**Raw sprats**	Microwave-assisted	0.03220	0.03205	0.03289	0.0324 ± 0.0005	12 mL extractant; vortexing for 10 s; 60 °C for 2 min with a 1 min ramp

Note: For each matrix–procedure combination, three separate analytical aliquots of the corresponding pooled homogenate were weighed and extracted separately. Results are expressed as mean ± sample SD (n = 3 analytical extractions). All extraction procedures used 0.15% (v/v) 2-mercaptoethanol in 0.01 mol/L HCl. Approximately 0.6 g of wet homogenised sample was used for each extraction. Total Hg in the resulting extracts was determined by ICP-MS, and the extracted-Hg values were normalised to dry weight using the measured dry matter content. Ultrasound-assisted extraction was selected for routine mercury-speciation analysis because it provided extracted-Hg concentrations comparable to rotational shaking in baked sardines and boiled sprats, a higher mean concentration in raw sprats, and a substantially shorter extraction time. The comparison reflects relative extraction performance and should not be interpreted as an absolute extraction-recovery study. [1]dw – Dry weight.

Table S5: ICP-MS conditions used to evaluate the relative extraction performance of the tested procedures.

Parameter	Condition
**Instrument**	Agilent 8900 ICP-MS (Agilent Technologies Inc., Santa Clara, CA, USA)
**Operating mode**	NoGas mode
**Extract dilution**	Dilution with 0.15% (m/v) L-cysteine in 1% (v/v) HNO_3
**Filtration**	0.22 µm pore-size filter
**Calibration range**	0.01-10 µg/L Hg
**Calibration standard**	Aqueous Hg standard solution, 1000 mg/L
**Calibration medium**	1% (v/v) HNO_3 containing 0.10% (m/v) L-cysteine
**Monitored isotope**	^{202}Hg
**Internal standards**	Luthetium (Lu) and bismuth (Bi), each at 50 µg/L

Figure S1: Representative HPLC-ICP-MS chromatogram of mercury species in baked sardines

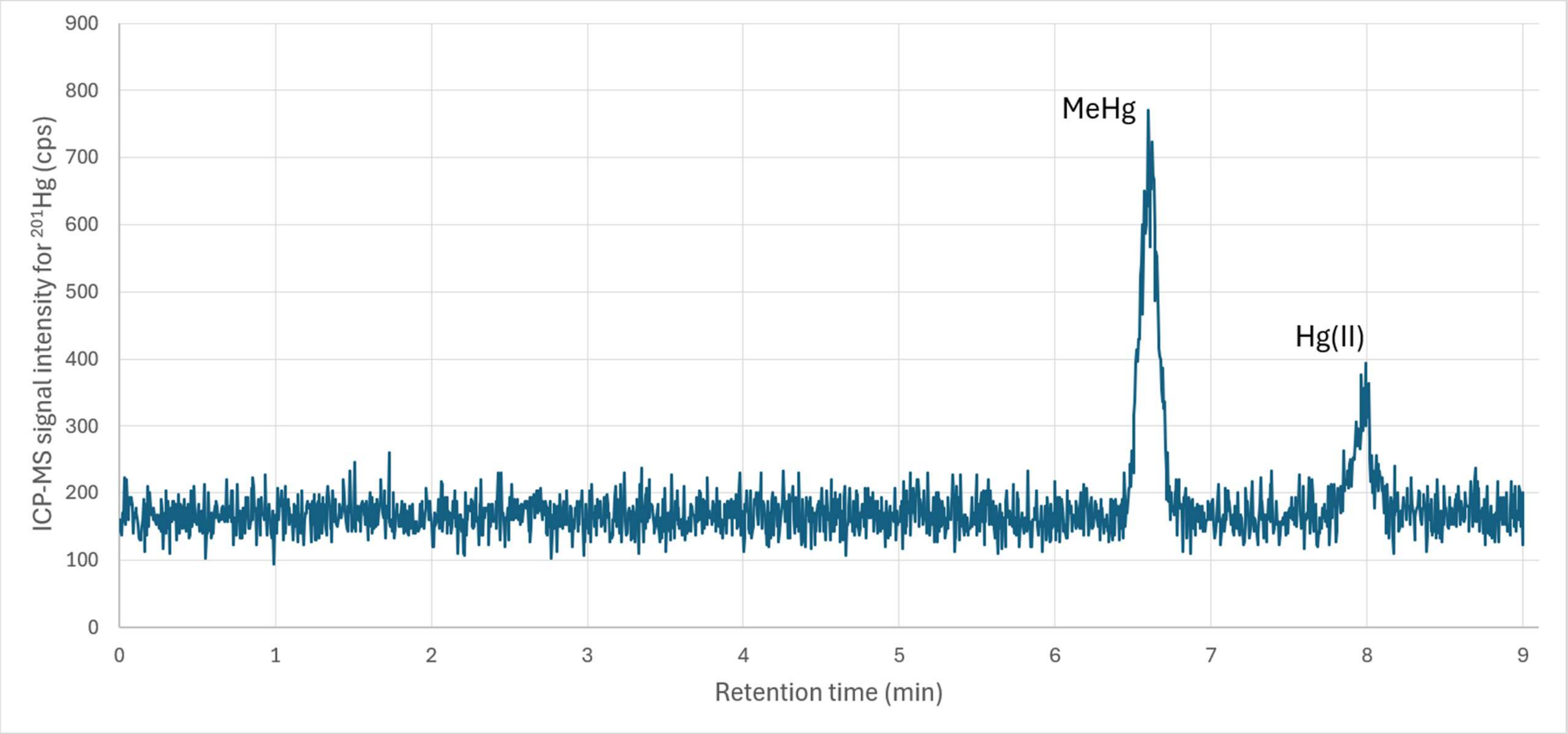

Note: Representative HPLC–ICP-MS chromatogram of mercury species in baked sardines, showing peaks assigned to MeHg and Hg(II). Peak identities were assigned by comparison with mercury-species standards, with retention times of 6.61 min for MeHg and 7.96 min for Hg(II). The ICP-MS signal was monitored at m/z 201. Quantitative mercury-speciation results for all analysed samples are presented in Table S3.
